# The Impact of Iodine Status on the Recall Rate of the Screening Program for Congenital Hypothyroidism: Findings from Two National Studies in Iran

**DOI:** 10.3390/nu9111194

**Published:** 2017-10-30

**Authors:** Ladan Mehran, Shahin Yarahmadi, Davood Khalili, Pantea Nazeri, Hossein Delshad, Zahra Abdollahi, Nasrin Azhang, Fereidoun Azizi

**Affiliations:** 1 Endocrine Research Center, Research Institute for Endocrine Sciences, Shahid Beheshti University of Medical Sciences, 1985717413 Tehran, Iran; lmehran@endocrine.ac.ir (L.M.); delshad1336@yahoo.com (H.D.); 2 Endocrinology and Metabolic Office, Center for Disease Control, Ministry of Health and Medical Education, 1419943471 Tehran, Iran; drshyarahmadi@yahoo.com (S.Y.); nasrinazhang@yahoo.com (N.A.); 3 Prevention of Metabolic Disorders Research Center, Research Institute for Endocrine Sciences, Shahid Beheshti University of Medical Sciences & Department of Biostatistics and Epidemiology, Research Institute for Endocrine Sciences, Shahid Beheshti University of Medical Sciences, 1985717413 Tehran, Iran; dkhalili@endocrine.ac.ir; 4 Family Health Institute, Breastfeeding Research Center, Tehran University of Medical Sciences, 1419943471 Tehran, Iran; pnazeri@tums.ac.ir; 5 Nutrition Office, Iran Ministry of Health, Treatment and Medical Education, 1419943471 Tehran, Iran; abdollaahi_z@yahoo.com

**Keywords:** iodine deficiency, congenital hypothyroidism, thyroid stimulating hormone, screening

## Abstract

Back ground: Iodine deficiency is one of the important factors in increasing the recall rate in congenital hypothyroidism (CH) screening programs. The present study assessed whether the iodine status of the general population may predict the recall rate or vice versa. Methods: In the current national study, among 1,382,229 live births delivered between March 2010 and March 2011, 1,288,237 neonates were screened for detecting CH by TSH (thyroid stimulating hormone) measurement via heel prick sampling. Simultaneously, a total of 11,280 school-aged children, aged 7–8 years, were selected using random multi-cluster sampling for measurement of urinary iodine. Results: A negative correlation was found between median urinary iodine (MUI) and the recall rate (*r* = −0.33, *p* = 0.03). No correlation was found between MUIC (median urinary iodine concentration) and the incidence rate of CH. Linear regression analysis showed a 0.1% increase in the recall rate for a one unit decrease in MUIC (β = −0.11, 95% CI: −0.2, −0.1, *p* = 0.03). MUIC, at a cut-off point of 144.7 µg/L, was predictive for a recall rate < 3% (*p* = 0.05). Conclusion: Frequencies of TSH ≥ 5 mU/L may be a more sensitive indicator for iodine status during pregnancy rather than in the general population. As higher recall rates reflect inadequate iodine nutrition, sufficient iodine supplementation is needed to reduce the recall rate in such communities.

## 1. Introduction

Iodine is an essential micronutrient for thyroid hormone synthesis [1]. Adequate iodine intake is required to sustain normal thyroid function and body metabolism, especially in pregnancy, during which maternal iodine requirements are increased due to increased thyroid hormone production, iodine transfer to the fetus (iodine in early pregnancy and thyroid hormones in later pregnancy), and increased urinary iodine loss [2]. Iodine deficiency in pregnant women can lead to psycho-neurological impairments in their neonates [3,4]. 

Iodine deficiency is one of the important factors in the increasing recall rate (the percentage of screening tests with elevated TSH) and the incidence of transient congenital hypothyroidism and infants born in iodine depleted areas tend to have higher thyroid stimulating hormone (TSH) concentrations as an adaptive mechanism to maintain serum thyroxin (T4) within normal or low normal ranges (128.7 to 205.9 nmol/L) [5]. One sensitive method to assess and monitor population iodine status is the prevalence of elevated TSH derived from the results of routine neonatal TSH screening [6,7]. Based on the World Health Organization (WHO) recommendation, the frequency of neonatal TSH > 5 mU/L in whole blood of <3% of the population indicates iodine sufficiency and frequencies of 3.0–19.9, 20.0–39.9, and >40.0% indicate mild, moderate, and severe iodine deficiency, respectively [6]. In Iran and some parts of the world [8,9,10], since the TSH cut off in the national screening program for congenital hypothyroidism has been set at 5 mU/L, frequencies of TSH values > 5 mU/L yield the recall rate in these areas. 

Data shows that in the past two decades, the prevalence of congenital hypothyroidism (CH) has been increasing worldwide [11,12], being even higher in some parts of the Middle East, especially in Iran [13,14,15], with a prevalence of 1.1–2.4 in 1000 births, compared to worldwide statistics of 1:3000–4000 [16]. In Iran the screening program for congenital hypothyroidism is performed by TSH measurement via heel prick blood sampling 3–5 days after birth, and neonates with TSH values over 5 mU/L are recalled for more confirmatory tests. Despite the overall success of screening programs in detecting congenital hypothyroidism, problems such as high recall rate and false positive results are still challenging issues, particularly in Iran, which may be due to inadequate iodine nutritional status [17,18]. Iran’s national elimination program for iodine deficiency disorder (IDD), launched in 1994 and evaluated every 5 years, indicates iodine sufficiency [17]; although, the median urinary iodine concentration (MUIC) has shown a decreasing trend over time, and some recent reports indicate iodine deficiency in parts of Iran [18]. A national study also demonstrated iodine deficiency in pregnant women despite sufficiency in the iodine status of the total population [19]. On the other hand, despite successful implementation of universal salt iodization programs over the last three decades, iodine deficiency remains a public health problem in many parts of the world due to a lack or lapse in the program’s sustainability; the major concern is its recurrence in countries believed to have achieved iodine sufficiency [20,21]. There are also reports of iodine deficiency in pregnant women living in iodine-sufficient areas, possibly because in addition to higher iodine requirement during pregnancy, diet-conscious pregnant women may avoid iodine-supplemented salt [22,23] despite their higher daily iodine requirement of 250 µg/day.

Considering the high prevalence of congenital hypothyroidism and the recall rate in Iran, and also considering reports of iodine deficiency in pregnant women, this study assessed the association of iodine status based on the median urinary iodine and recall rate and the CH incidence in all provinces in Iran based on the results of two national studies conducted in 2011.

## 2. Methods

### 2.1. Thyroid Stimulating Hormone of Neonates in National Screening Program for CH

In Iran, medical education, research, and health services are all integrated and supervised by the Ministry of Health and Medical Education and health care in all provinces is under coverage of the universities of medical sciences in each province. All 46 universities from 31 provinces were included in this study. Among 1,382,229 live births delivered between March 2010 and March 2011, 1,288,237 neonates were screened for detecting CH. Screening was performed by TSH measurement on a filter paper blood spot by heel prick sampling, obtained 3–5 days after birth. Neonates with a Guthrie TSH test ≥ 5 mU/L, were recalled for confirmatory tests (with venous blood samples) and the final diagnosis was made by a pediatric endocrinologist, appointed as the focal point of the CH program in each university region. Neonates with TSH > 10 mU/L and T4 < 6.5 µg/dL in venous samples were diagnosed as having hypothyroidism [24] and promptly treated by a physician. Central data regarding birth rate and number of CH subjects were collected from each health region and sent to the Research Institute for Endocrine Sciences (RIES), Shahid Beheshti University of Medical Sciences. TSH levels in screening samples were measured by enzyme-linked immuno-sorbent assay (ELISA) using Iran Padtan Elm Company kits, and serum levels of TSH and T4 were measured by immune-radiometric assay (IRMA) and radioimmunoassay (RIA) methods, with Kavoshiar diagnostic kits (Tehran, Iran), respectively. 

### 2.2. Urinary Iodine Concentration of School-Aged Children in National Monitoring Program for IDD

Simultaneously (2010–2011), data on population iodine status were extracted from the national monitoring program for elimination of iodine deficiency under the supervision of the Nutrition Office, Ministry of Health and Medical Education. A total of 11,280 school-aged children (240 from each region) aged 7–8 years were selected from different schools in each region using random multi-cluster sampling [25]. Briefly, in each university district, 20 codes of the households of 10 female and 10 male children were randomly selected and by face to face questioning their school addresses were obtained. From each subject’s school, 2 clusters, each containing 6 schoolchildren based on the child’s school code, were selected. Thus, 40 clusters containing 240 schoolchildren (120 rural and 120 urban) were enrolled from each university district, resulting in 11,280 subjects as the total sample size of the present study.

Spot urine samples (10 mL) were collected between 08.00 and 11.00 am, while children attended school and examined in the reference laboratory in each province for iodine measurement, based on the WHO protocol. Since morning fasting urine generally tends to underestimate the iodine status, non-fasting spot urine samples can be taken randomly at any time during the day [26]. Urinary iodine was measured by acid digestion method based on a modification of the Sandell-Kolthoff reaction. 

The ethics committee of the Research Institute for Endocrine Sciences of Shahid Beheshti University of Medical Sciences approved the protocol for this study (ethic code: IR.SBMU.ENDORINE.REC.1395.341) and written informed consent was obtained from the parents. 

### 2.3. Definition of Terms

The recall rate is the percentage of screening tests with elevated TSH (≥5 mU/L), based on which the physician notifies the authorities to contact the parents in order to arrange another test [7,27]. According to criteria of the WHO, International Council for the Control of Iodine Deficiency Disorders (ICCIDD) and the United Nations Children’s Fund (UNICEF), MUICs < 20, 20–49.9, 50–99.9 and ≥100 µg/L in school-aged children represent severe, moderate, and mild iodine deficiency and iodine sufficiency, respectively [6].

### 2.4. Statistical Analysis

Median urinary iodine concentration, recall rate, and incidence rate of congenital hypothyroidism in each province were tested for normality using Shapiro-Wilk tests (*p* = 0.304). In order to investigate the association between MUIC and indices of congenital hypothyroidism, assuming normality, a Pearson product-moment correlation was run between MUIC, recall rate, and incidence rate. All statistical assumptions were confirmed using scatter plots. An independent *t*-test was also used to determine whether there was any statistical difference in means of MUIC in provinces with recall rates > 3%, in comparison to those with recall rates < 3%. Assumption of homogeneity was confirmed using Levene’s Test (*p* = 0.163). The receiver operating characteristic (ROC) curve was calculated in order to find the optimal threshold (best cut-off point) of MUIC in prediction of recall rate. *p* values < 0.05 were considered significant. All statistical analyses were performed using SPSS 15.0 statistical software package (SPSS, Chicago, IL, USA).

## 3. Results

Frequencies of neonatal TSH of heel prick sampling, recall rates, and incidence rates of congenital hypothyroidism in national screening program by university region are summarized in Table 1. On a national level, screening revealed TSH < 5, 5–9, 10–19.9 and ≥20 mU/L in 97.2, 2.6, 0.2, and 0.0% of neonates, respectively; as shown, recall rates range from 0.2% to 6.0%. Also, the incidence rate of CH vary from 1 in 196 to 1 in 2316 births.

Median urinary iodine values and percentage of urinary iodine < 20, 20–49.9, 50–99.9, 100–299.9, and ≥300 µg/L in schoolchildren of each region are presented in Table 2. On the national level, urinary iodine < 20, 20–49.9, 50–99.9, 100–299.9, and ≥300 µg/L was found in 1.9, 5.7, 16.2, 66.0, and 10.1% of school-aged children, respectively, indicating the highest and lowest values of MUIC to be 270.0 and 76.0 µg/L, respectively.

There was a high correlation between recall rate and incidence rate of CH (β = 0.7, *p* < 0.001). MUIC was correlated with TSH screening values of 5–9.9 (*r* = −0.32, *p* = 0.03) and ≥10 mU/L (*r* = −0.56, *p* < 0.001). Also, MUIC was significantly lower in provinces having recall rates ≥ 3% compared to those with recall rates <3% (134.0 µg/L vs. 164.0 µg/L, *p* = 0.03). 

A negative linear correlation was found between median urinary iodine levels and recall rate (*r* = −0.33, *p* = 0.03); however, the nonlinear quadratic correlation showed a significant improvement in *R*^2^ with a coefficient of −0.48 for X and +0.01 for X^2^. There was no correlation between MUIC and incidence rate of congenital hypothyroidism. 

Recall rate was higher in regions with MUIC <100 µg/L compared to regions with MUIC ≥ 100 µg/L, (4.1% vs. 2.5%, *p* = 0.05). Our results indicate that 11 out of 46 regions (23.9%) had a recall rate ≥ 3%, while, MUIC < 100 µg/L was observed in 3 out of 46 regions (6%). Linear regression analysis also showed a 0.1% increase in recall rate for each unit decrease in MUIC (β = −0.11, 95% CI: −0.2, −0.1, *p* = 0.03).

**Figure 1:**
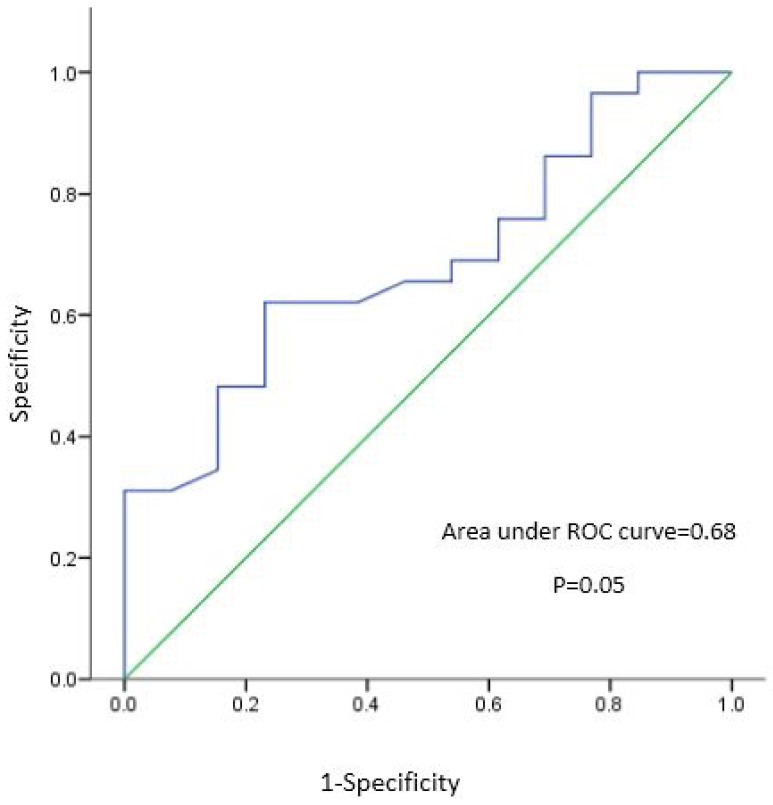
Receiver-operating characteristic (ROC) curve and corresponding area under the curve (AUC) statistics for probability of detection recall rate < 3 vs median urinary iodine cut-offs.

As shown in Figure 1, ROC curve analysis demonstrated the median urinary cut off point of 144.7 µg/L to be predictive for recall rates < 3%, with sensitivity 62% and specificity 77% with an area under curve of 0.68 (*p* = 0.05).

## 4. Discussion

To the best of knowledge, this national study is one of the first to investigate whether iodine status in a general population (defined by the MUIC values in school-aged children), in an area with iodine sufficiency, can be correlated to the recall rates and incidence rates of congenital hypothyroidism in newborns or vice versa. Findings of the current study indicate a weak negative association between median urinary iodine concentrations in school-aged children and the recall rate in the newborn screening program for CH, demonstrating that higher recall rates reflect inadequate iodine status; however, no association was found between urinary iodine levels and incidence of CH. 

The distribution of neonatal TSH concentration, along with median urinary iodine of school-aged children, are reported to be sensitive indicators of population iodine status [6,7,27]. Median urinary iodine of school-aged children <100 µg/L and frequencies of neonatal TSH > 5 mU/L in whole blood in over 3% of neonates indicate iodine deficiency. On a population basis, there is a negative association between urinary iodine concentration and neonatal TSH values, specifically in countries with moderate to severe iodine deficiency [28]; however, in countries with mild to borderline iodine deficiency, decline in UIC is not accompanied by change in blood spot TSH concentration [28]. In the current study, since these two indicators were not always compatible in most regions, the association found between MUIC in school-aged children and neonatal TSH concentrations >5 mU/L was not a strong one.

Based on previous reports, in Iran the rate of recall and incidence of CH are higher than those of other countries [29]. High recall rate imposes a heavy burden on the screening program via project expenses, a heavy work load for staff, and stress on families, which is why determining its associated factors in order to reduce recall rate of the program is a major issue of concern. High recall rates may be due to multiple factors, e.g., different screening strategies (use of T4 or TSH or both), different laboratory techniques, site of sample collection, iodine status, different recall criteria, and human error [30,31,32,33]; of these factors, iodine deficiency seems to be of higher concern, especially in Iran. Universal salt iodization has been successfully implemented in Iran since 1996 [34] and sustainability of the program based on monitoring surveys every 5 years is regularly reported; yet, the trend of MUIC is decreasing, although still within the sufficient limit [17,35]; national MUICs were reported to be 232, 190, and 140 µg/L in 1996, 2001, and 2006, respectively. There is also some data on recurrence of iodine deficiency in some parts of Iran [18]. Reports of a national study are indicative of iodine deficiency in pregnant women (MUIC: 87.3 µg/L) despite data on iodine sufficiency in school-aged children [19]. As maternal iodine nutritional status affects neonatal TSH concentrations, the reported iodine deficiency in pregnant women in previously presumed iodine-sufficient areas may justify the discrepancy observed between MUIC in school-aged children and neonatal TSH values. In this survey, 11 of 46 regions (23.9%) had recall rates > 3%, while only 3 of 46 regions (6%) had MUIC < 100 µg/L. The American Thyroid Association and Endocrine Society recently recommended a daily iodine intake of 250 µg for all pregnant women living in both iodine-deficient and -sufficient areas [36], advice which may reduce this discrepancy between these two indicators and recall rates. The current findings are highlighted by reports from other countries where iodine deficiency has been observed in pregnant women residing in iodine-sufficient regions, e.g., USA [37,38], or even in Japan, which is regarded as a country with iodine sufficiency or even excessive iodine intakes [39]. 

In addition, in our iodine-sufficient area, no association was observed between MUIC and incidence of CH, which may be due to the difference between MUIC in school-age children and MUIC in pregnant women (not measured). Other factors which affect the incidence of CH should also be considered, e.g., maternal TSH receptor-blocking autoantibodies, exposure to excess iodine, use of goitrogenic agents or antithyroid drugs during the perinatal period, very low birth weight, and prematurity and genetic factors, not just inadequate iodine status per se [27,40].

The main strengths of this study are assessment of the impact of iodine status at population level on the recall rate and incidence of congenital hypothyroidism, for the first time in an area of iodine sufficiency. The results derived from two national surveys facilitated data analysis based on population indicators and not individual data. The limitations, however, include small sample size (areas), especially a few areas with MUIC < 100 µg/L and using a single urine sample due to significant day to day variations in salt intake (the main source of dietary iodine in Iran), and also variations in hydration in individuals [41,42]; on the other hand, using heel blood sample to estimate recall rate and incidence of CH may be less sensitive to iodine status compared to cord blood samples [43]. Another limitation is not having data regarding thyroglobulin, which has been known as another indicator of iodine deficiency [44], to compare the results with UIC and neonatal TSH and lack of data of iodine status assessment in pregnant women, which may correlate better with the recall rate of newborn thyroid screening or incidence of CH.

## 5. Conclusions

In conclusion, although both MUIC and the neonatal TSH distribution have been introduced as indicators of the iodine nutritional status of population; moderate association and discrepancies between neonatal TSH levels and MUICs in school-aged children were found in our study, suggesting that the percentage of TSH > 5 mU/L may be a more sensitive indicator for maternal iodine status during pregnancy than iodine status of the general population, specifically due to evidence of iodine deficiency among pregnant Iranian women; hence, prescribing prenatal supplements containing 150 µg iodine is warranted to reduce the recall rate in screening program for congenital hypothyroidism in Iran. More community-based studies with larger sample sizes need to be conducted to define more sensitive cut-points for MUIC of the population to precisely predict recall rate for the congenital hypothyroidism screening program. 

## Figures and Tables

**Table 1:** Frequency of neonatal thyroid stimulating hormone, recall rate, and incidence of congenital hypothyroidism *.

Region	Neonatal Thyroid Stimulating Hormone mU/L (%)				Recall Rate (%)	Incidence Rate (*n*)
	<5	5–9	≥10	≥20		
Ahvaz	95.8	3.7	0.4	0.1	4.2	1/350
Alborz	97.8	1.8	0.3	0.1	2.2	1/621
Ardebil	99.0	1.0	0.03	0.1	1.0	1/921
Azaebaijan, West	97.4	2.4	0.2	0.1	2.6	1/575
Babol	98.3	1.6	0.1	0.1	1.7	1/1120
Bam	97.6	1.9	0.1	0.4	2.4	1/512
Charmahal Bakhtiari	97.4	2.3	0.2	0.1	2.6	1/360
Dezful	95.8	3.7	0.4	0.2	4.2	1/196
Fasa	97.9	2.1	0.02	0.0	0.2	1/820
Ghazvin	97.4	2.3	0.2	0.0	2.6	1/345
Ghom	94.3	5.5	0.2	0.1	5.7	1/359
Gilan	99.0	0.9	0.1	0.1	1.0	1/610
Golestan	98.2	1.6	0.1	0.1	1.8	1/620
Gonabad	98.2	1.8	0.0	0.0	1.8	1/719
Hamedan	98.7	1.2	0.1	0.1	1.3	1/793
Hormozgan	97.2	2.7	0.1	0.1	2.8	1/604
Ilam	98.2	1.7	0.1	0.1	1.8	1/343
Isfahan	98.4	1.4	0.1	0.1	1.6	1/532
Jahrom	99.6	0.4	0.03	0.0	0.4	-
Jiroft	98.3	1.6	0.1	0.1	1.7	1/644
Kashan	98.5	1.3	0.1	0.0	1.5	1/536
Kerman	97.0	2.9	0.1	0.0	3.0	1/446
Kermanshah	98.7	1.2	0.1	0.1	1.3	1/839
Khorasan, North	95.0	4.5	0.4	0.0	5.0	1/429
Khorasan, South	94.8	4.9	0.2	0.1	5.2	1/325
Kohkiloyeh	98.6	1.1	0.2	0.1	1.4	1/402
Kordestan	95.5	4.2	0.3	0.0	4.5	1/307
Lorestan	94.9	4.6	0.4	0.1	5.1	1/272
Markazi	96.8	3.0	0.1	0.0	3.2	1/305
Mazandaran	97.6	2.2	0.1	0.1	2.4	1/747
Neishaboor	97.3	2.6	0.1	0.1	2.7	1/926
Rafsanjan	94.7	5.8	0.1	0.1	6.0	1/196
Razavi Khorasan	96.8	3.0	0.1	0.1	3.2	1/617
Sabzvar	96.5	3.1	0.2	0.1	3.5	1/313
Semnan	98.4	1.4	0.1	0.1	1.6	1/513
Shahrood	97.9	1.9	0.1	0.0	2.1	1/2316
Shahid Beheshti & Iran	95.8	3.7	0.4	0.1	4.2	1/434
Shiraz	98.2	1.6	0.1	0.0	1.8	1/540
Tabriz	97.4	2.3	0.2	0.1	2.6	1/593
Tehran	97.2	2.3	0.4	0.1	2.8	1/490
Torbat Heidarieh	98.6	1.3	0.1	0.0	1.4	1/1130
Zabol	98.8	1.1	0.1	0.0	1.2	1/618
Zanjan	95.9	3.9	0.2	0.0	4.1	1/259
Zahedan	98.0	1.7	0.2	0.1	2.0	1/326
Yazd	98.1	1.8	0.1	0.0	1.9	1/482
National	97.2	2.6	0.2	0.0	2.8	1/465

* All newborns from university regions, including 1,288,237, neonates were included.

**Table 2:** Median urinary iodine concentration (MUIC) in school-aged children in national monitoring program for iodine deficiency by university of region.

Region		MUIC, µg/L (%)				
		<20	20–49.9	50–99.9	100–299.9	≥300
Ahvaz	104.0	17.1	10.8	20.0	35.0	17.1
Alborz	122.5	0.0	11.1	26.1	59.8	2.9
Ardebil	150.0	0.0	0.0	11.2	86.3	2.5
Azaebaijan, West	90.0	0.4	17.0	30.0	36.2	12.5
Babol	230.0	0.0	0.0	3.6	72.8	23.6
Bushehr	117.6	0.0	7.3	32.4	58.0	2.3
Charmahal Bakhtiari	192.0	1.6	0.4	12.3	68.5	17.2
Dezful	142.1	0.0	0.8	5.0	90.2	4.0
Fasa	140.0	0.0	2.9	12.2	82.5	2.2
Ghazvin	140.2	0.0	3.6	12.7	60.0	23.6
Ghom	143. 0	0.0	2.1	22.9	68.3	6.7
Gilan	202.0	0.0	0.0	10.8	61.3	27.9
Golestan	132.6	5.7	16.7	20.8	46.1	10.6
Gonabad	189. 0	1.3	0.0	3.4	87.3	8.0
Hamedan	134.0	0.4	9.6	24.3	60.0	5.8
Hormozgan	203.3	0.8	4.3	7.6	59.0	28.3
Ilam	121.5	1.7	6.7	27.9	56.3	6.7
Isfahan	155.0	2.9	5.9	20.6	57.1	13.4
Jahrom	200.0	0.4	2.5	9.6	83.3	4.2
Jiroft	210.0	0.0	5.4	10.8	54.6	29.2
Kashan	137.0	1.2	8.3	22.7	60.8	7.0
Kerman	252.5	0.4	2.6	3.5	44.3	49.1
Kermanshah	146.5	6.5	8.6	19.5	50.8	14.6
Khorasan, North	140.0	0.8	12.1	29.9	53.0	4.2
Kohkiloyeh	194.0	0.5	4.9	9.3	68.3	16.9
Kordestan	76. 0	3.2	36.9	19.4	35.7	4.8
Lorestan	80.0	32.0	6.0	21.0	37.0	5.0
Markazi	190.0	0.4	4.6	7.9	75.4	11.7
Mazandaran	126.0	0.0	0.0	6.9	93.1	0.0
Neishaboor	153.0	0.0	4.8	14.3	75.2	5.2
Rafsanjan	136.5	0.0	4.8	20.0	68.8	6.4
Razavi Khorasan	103.0	0.0	2.5	23.5	67.8	6.2
Sabzvar	189.0	2.0	1.2	4.0	82.1	10.5
Semnan	117.0	3.3	9.2	26.4	47.5	13.3
Shahrood	270.0	0.0	5.4	13.4	68.2	13.0
Shahid Beheshti & Iran	139.2	0.0	3.3	24.2	65.0	7.5
Shiraz	172.0	2.2	7.1	11.1	67.1	12.4
Tabriz	109.0	0.0	2.1	39.2	56.6	2.1
Tehran	114.2	2.0	8.0	25.4	63.3	1.3
Torbat Heidarieh	160.0	3.3	7.1	18.8	47.6	2.3
Zahedan	177.8	0.0	0.0	6.8	86.8	8.4
Zanjan	157.0	0.0	5.0	29.0	62.3	3.7
Yazd	176.0	0.0	2.0	15.0	73.0	10.0
National	144.7	1.9	5.7	16.2	66.0	10.1
